# Students and tutors' social representations of assessment in problem-based learning tutorials supporting change

**DOI:** 10.1186/1472-6920-9-30

**Published:** 2009-06-07

**Authors:** Valdes R Bollela, Manoel HC Gabarra, Caetano da Costa, Rita CP Lima

**Affiliations:** 1 Medical Education Department, Universidade Cidade de São Paulo (UNICID) School of Medicine, São Paulo, Brazil; 2 Medical Informatics Department, Universidade de Ribeirão Preto School of Medicine, Ribeirão Preto, Brazil; 3 School of Medicine, Universidade de Ribeirão Preto (UNAERP), Ribeirão Preto, Brazil; 4 Education Department. Universidade Estácio de Sá, Rio de Janeiro, Brazil

## Abstract

**Background:**

Medical programmes that implement problem-based learning (PBL) face several challenges when introducing this innovative learning method. PBL relies on small group as the foundation of study, and tutors facilitate learning by guiding the process rather than teaching the group. One of the major challenges is the use of strategies to assess students working in small groups. Self-, peer- and tutor-assessment are integral part of PBL tutorials and they're not easy to perform, especially for non experienced students and tutors. The undergraduate PBL medical programme was introduced in 2003, and after two years the curriculum committee decided to evaluate the tutorial assessment in the new program.

**Methods:**

A random group of ten students, out of a cohort of sixty, and ten tutors (out of eighteen) were selected for semi-structured interviews. The social representations' theory was used to explore how the students and tutors made sense of "assessment in tutorials". The data were content analyzed using software for qualitative and quantitative processing of text according to lexicological distribution patterns.

**Results:**

Even though students and tutors are aware of the broader purpose of assessment, they felt that they were not enough trained and confident to the tutorial assessment. Assigning numbers to complex behaviors on a regular basis, as in tutorials, is counter productive to cooperative group learning and self assessment. Tutors believe that students are immature and not able to assess themselves and tutors. Students believe that good grades are closely related to good oral presentation skills and also showed a corporative attitude among themselves (protecting each other from poor grades).

**Conclusion:**

Faculty training on PBL tutorials' assessment process and a systematic strategy to evaluate new programs is absolutely necessary to review and correct directions. It is envisaged that planners can make better-informed decisions about curricular implementation, review and reform when information of this nature is made available to them.

## Background

Problem-based learning (PBL) form the backbone of theoretical learning in some medical schools that advocate student-centered teaching methods. This approach stimulates students to play an active role in the learning process as compared to the passive information transmission, typical of traditional teaching methods. PBL relies on small group as the foundation of study, and tutors facilitate learning by guiding the group process rather than teaching the group[1,2]. Medical programmes that implement PBL face several challenges when introducing this innovative teaching and learning method. One of the major challenges is the use of appropriate strategies to assess students working in small groups and to train faculty and students to run formative assessment as an integral part of students' assessment in the horizontal and vertical modules of the program[3]. A key educational concern is how to assess students comprehensively and fairly using self-assessment, peer assessment and tutor assessment, as these new practices often make tutors and students anxious[4,5]. This complex process is affected by personal and interpersonal perceptions, which produces and results from a shared view of reality. The discourse produced by students and tutors in this study conveys perceptions about assessment which are formed socially and contains symbols and subjectivity that are collectively constructed, as proposed by Moscovici in 1961, in the social representations' theory [6].

Moscovici showed the importance of the studies done in 1898 by the philosopher Durkheim on the theory of social representations, who suggested that there is a distinction between the world of common sense, including social representations, and the concrete world of science[7]. Social psychology postulates that: normal individuals react to phenomenon like scientists do and "understanding" consists in information processing. We are often unaware of things before our eyes and we make similar conclusions of reality based on our social information. Our reality is based on social representations, which conventionalize objects, persons, and events we encounter. Each experience is added to a reality predetermined by conventions. Individuals and groups create representations in the course of communication and co-operation. Representations are born, change, and change other representations. The task of social psychology is to study these representations. Social representations should be seen as a specific way of understanding and communicating what we know already. The actuality of something absent, the "not quite rightness" of an object, are what characterize unfamiliarity. Representations help make the unfamiliar familiar. Social thinking owes more to convention and memory than to reason. Our tendency is to confirm what is familiar. One anchors the unfamiliar in the current conventions of reality. Objects are threatening until named. Anchoring is taking something foreign and compares it to a paradigm that might be suitable. When we compare unfamiliar things to a prototype, we notice those things most representative of the prototype. Naming something locates the object in an identity matrix. Naming something give it certain characteristics and tendencies. Classifying and naming anchor representations. The main object of representations is to help interpretation, understanding, and opinion formation[6,7]. Social representation is a growing field that has continued to attract new researchers from across Europe, South America, Australia and even the USA over the last 40 years[8,9].

This study aimed to explore how students and tutors make sense (social representations) of the "assessment in PBL tutorials", and how the results might lead to curriculum evaluation and improvement.

## Methods

### Context of study

The undergraduate PBL medical programme at the University of Ribeirão Preto was introduced in 2003. The programme is organized into eight semesters. There are three modules per semester. Each module lasts six to seven weeks and eight problems are addressed per module. Each small group, comprising 10 students, meets twice a week. Assessment takes place at the end of every opening and closing session for each problem, and six criteria are used by both students and tutors to assess student performance using a 10-point rating scale. These criteria are related to the abilities: to ask questions; to use previous knowledge; to use new knowledge; to formulate hypotheses; to integrate knowledge related to the problem; to provide and receive feedback. PBL assessment means 40% of the module grade and is made up of three components: 60% tutor assessment, 20% peer assessment, and 20% self-assessment.

### Participants

A random group of 10 students, from a cohort of 60 students (the first cohort of students that started the PBL programme in 2003) were selected when they were doing their fourth semester class. Students were selected randomly among those that had completed the first, second and third semesters of the PBL curriculum. The choice for students from the first batch was due to the fact that they have experienced the curricular change and the implementation of the new methodology more closely. Ten out of eighteen tutors who had taught in the first semester class were randomly selected to the interviews too.

### Data gathering

Semi-structured one-on-one interviews explored the same topics in both groups: opinions regarding the PBL tutorials, about learning process and the role of assessment in them. Informed consent was obtained from each participant and confidentiality was ensured. All interviews were recorded, transcribed and reviewed by participants prior to inclusion in the study.

### Text analyses

The data were subjected to content analysis using ALCESTE (Analyze Lexicale par Contexte d'un Ensamble de Segments de Texte) software. This programme facilitates qualitative and quantitative analysis of text data according to lexicological distribution patterns. This provides a perspective that brings order and coherence to the topic of discourse. The following analyses were conducted on the content of each interview: an accurate transcription of tapes was made, the transcription was revised and adapted to the standards of the ALCESTE software, which aided the analyses of textual materials. The program offers a table that presents the distribution of the classes formed and allows a primary compression of the obtained classes, mainly by the vocabulary (lexicons) [10,11]. When texts produced by a number of individuals are studied, the goal is to understand the view that is collectively shared by that social group at a given time. In all interviews the goal was to analyze what students and tutors said in relation to "assessment in PBL tutorials" and how they combined these elements.

The type of assessment under investigation in this study takes account of the interrelationship between experiences and emotions, that is, the manner in which students and tutors assign meaning as a result of the interaction between cognitive and affective components. Even though judgment based on values tends to be present in this type of assessment, the relevance of consolidating an assessment culture which critically monitors educational practices was also observed. The analysis of students and tutors' discourse from the perspective of social representations theory sheds light on some complex issues regarding assessment in PBL tutorials.

### Ethics issues

Students and tutors were asked to give verbal informed consent. Their identities were not disclosed during the study. This protocol was approved by CEP-UNAERP (Research Ethical Board) registered under the number ComEt: 061/04.

## Results

The qualitative analysis identified categories of data relevant to assessment in PBL tutorials. Two categories were identified for students: assessment difficulties and the role of feedback and self-assessment. Two categories were identified for tutors: assessment difficulties and the role of tutors in student assessment.

### The discourse of students

The main categories identified in the students' discourse were "assessment: difficulties" and the role of feedback and self-assessment. Their answers show how learning mechanisms and strategies operate in relation to assessment practices. The difficulties concerning assessment practices in tutorials were a recurring theme, strongly linked to the process of grading performance. For example:

"... nine is excellent... and below five no one knows how to grade... no one knows the meanings of two, three or four..."

"... that was an eight for "expression", a five for "study" and a ten for something else.... so, I guess, either cut down on the 10-points scale or include another criterion: sufficient, insufficient, fair...".

Assessment in tutorials requires that students be capable of providing and receiving critical feedback as well as performing self assessment. This often resulted in students feeling insecure and uncomfortable. This not only reveals the subjective nature of assessment, but also the affective factors that lead to specific attitudes, like corporative issues between students. For example:

"... then I won't give him a bad grade... how can you know that he is good? You can't... there's the label..."

"... because you begin to win a kind of cooperation among people, it's hard to give them a poor grade... you do that when it's really critical."

Some answers reveal the interrelationship between assessment, individual traits and behavior present in tutorials. Once again, the issue of grades was influential in determining behavior and attitudes[12], particularly the concern that good grades are dependent upon good oral presentation skills. For example:

"... because I, for instance... I am a student who may study, but I don't like talking, I hate talking, I don't feel good talking... now you are forced to talk for two years to get grades... Then I end up talking, but I can't express what I really know, you know?"

"... well... it's been tough... because there's the one who talks the most, there's the one who talks the least, there are some halfway... Are you going to give a ten to those who talk the most, five or six to those who talks the least and you'll give a seven or eight to those in the middle?..."

Although assessment in PBL tutorials is aimed at making students think freely about their own learning process, it can be noticed that they still perceived the assessment process as a ranking tool. This created fear and anxiety despite repeated attempts to stress the long term commitment to learning rather than an exclusive focus on grades. As far as critical feedback is concerned, students tended to refer to the attitude of the tutor during tutorial sessions and the lack of criteria when grades were assigned. For example:

"... I think the way students are motivated needs to be evaluated... I've had a tutor who was unable to assess students and who made the whole tutorial session demotivating... and I've already had a tutor who spoke very correctly about each person, and therefore stimulated..."

"... now, the [tutor] I'm with, for instance, has a reputation for giving low grades, and that's what he actually does... The last [tutor] used to give high grades to the same people... I think that's wrong, absolutely wrong... there has to be some standardization... what has to be said is: 'the profile of student X corresponds to grade Y..."

In some answers, it was possible to recognize that the criticism of the assessment process is related to self-criticism. This suggests that students also play a challenging part in the process since they are not certain how to behave. For example:

"...that's tough too..." then you think: "will I give myself a high grade?..." "How well did I do?" "It's hard... self-assessment is hard... how do I assess?"

### The discourse of tutors

The discourse regarding assessment includes issues relevant to critical feedback, self-assessment and the role of tutors in the assessment process. As a whole, the data show that, in theory, tutors understand the goals of assessment. However, in practice they often use the traditional model of assessment. Nevertheless, tutors did try to reflect on their own practice and searched for new strategies regarding assessment practices.

One of the major challenges tutors faced was the difficulty of establishing objective criteria for assessing this type of student performance, especially as far as grading is concerned. For example:

"... what I have most difficulty with, and so do the students, is assessment... to know exactly the weight..."

"It would be necessary to specify each item and reach a consensus about what each item represents. ...For example, the ability to recognize, the ability to critically assess the information that is presented or the source of the information..."

"...because I also find it difficult sometimes to discriminate grade by grade, student by student..."

Some answers suggest that the issue of grades is also a source of concern for the tutors. By realizing the subjectivity of the assessment process, tutors also assumed that students were not using objective criteria for the purpose of peer and tutor assessment. For example:

"... students have this overall difficulty... if they have difficulty assessing their peers, they will also have difficulty assessing their tutors... so, if they are giving high grades to their tutors, they might be giving high grades to their peers as well..."

It should be noted that tutors are aware of the need for students to learn how to assess performance in PBL tutorials. For example:

"... many students are not mature enough..." "...there are students who simply shut their eyes and give ten to everybody".

The key role tutors play in assessment clearly emerges from the interview data. The power to grant grades is viewed as one of their important jobs. Even though they attempted to view assessment as a tool to pass and change students, they still demonstrate controlling attitudes that are typical of the traditional culture of assessment. For example:

"... so, it is a valuable tool, but we need to know how to use it... I rarely give a ten... In order for that to happened, I must feel astounded... zero, I don't give that... zero is only for those who were not present... the little experience that I have, that's the way it is...".

"... here you have to use more technical terms, more adequate... So, therefore, what ends up happening? I rarely give a nine or a ten in tutorial assessments in all the items... very seldom... Usually, my grades range, on average, between five and six..."

The tutor's decision-making power in this regard is central:

"...tutors, because of their influence on the group, find it easier to assess... they are in the position of privileged spectators watching the discussion..."

## Discussion

The overall purpose of assessment has undergone significant transformation over the past three decades. Previously the primary goal of assessment was to measure student performance in order to assign grades and make judgmental decisions. This was often done at the expense of recognizing the individual and the collective potential of students [13,14]. More recently, the role of assessment as a tool to facilitate learning has come to be recognized. Indeed, the stressful ritual of end-of-course examinations has been losing ground in favour of continual assessment and its role as part of the learning process [15].

It is important to recognize that the formulation of assessment reports based on experiences takes place in a subjective domain and are not simply personal or produced in isolation. The study explored how students and tutors made sense of assessment in PBL tutorials. Similarities between the discourses of students and tutors were observed, particularly when assumptions about a new assessment paradigm were at play, contradicting the traditional model of assessment where teachers are exclusively in charge of the assessment process.

Both students and tutors recognized that assessment is important but it is also an extremely hard task. Grading performance with a 10-point scale was perceived by students and tutors as a critical issue. Traditionally, most medical schools use grades instead of criteria to make decisions about students' progress. Assigning numbers to complex behaviors on a regular basis, as in tutorials, is counter productive to cooperative group learning and self assessment. To this point we had a suggestion from a student to change from 10-point scale grade to criterion referenced system (sufficient/insufficient) for the tutorials. This understanding was helpful to the medical school when, in 2006, they decided to change from 10-point scale to a criterion-referenced rating scale with only three descriptors (satisfactory, fair and unsatisfactory).

Students also felt uncomfortable to provide and receive feedback. We believe that it might be amplified in Latin cultures, where people usually perceive feedback as criticism and avoid using it. We also realized that students showed a corporative attitude among themselves (protecting each other from poor grades). This behavior was also observed in a study comparing tutor, self- and peer-assessment from 349 first year Brazilian students in PBL tutorials where tutors' marks were consistently lower than students' self- and peer-assessment marks[16]. On the other hand, a study in the United Arab Emirates following five year students showed that self- and tutors scores were similar but with male student self-assessment scores higher than for female on overall scores[17]. Another study in Australia with first year students showed that self-assessment on PBL tutorial results in substantial under-marking compared to tutor assessment, and peer-assessment's score were significantly more generous than those arising from tutor assessment[18]. Most of PBL schools report assessment during tutorials, but its purpose (summative or formative) is usually not obvious to students and faculty members. When stated, the use of assessment during tutorials is quite different, especially because it possesses psychometric shortcomings that limit their use in high-stake decision making. Despite the differences, there might be a change on the social and cultural meaning of assessment and in the practices so that students and teachers look to assessment as a source of insight and help, instead of an occasion for meting out rewards and punishments[14,19].

Students also believe that good grades are closely related to good oral presentation skills, and they would be graded according to the amount of information they say during tutorials. We observed that tutors' references to grade students were not well standardized and it was clearly recognized by students. Moreover, this should interfere in the reliability and would be perceived by students as an unfair process [20]. Tutors also believe that students are immature and not able to assess themselves and tutors, which seems to be a contradiction. Neither students nor tutors realized that developing skills to perform any kind of assessment and decision making is one of the aims to the small group dynamic and it is also a desirable skill to the future doctors' profile, as described in the pedagogic project of the Medical School.

Finally, a common concern among tutors was related to the uncomfortable relationship between a new teaching method and well-known traditional methodologies. Resistance and doubt was the result of conflict between what was traditional and what was new. In addition, tutors and students openly expressed concern regarding the lack of training and experience in the process of assessing performance in PBL tutorials. A trend towards finding strategies to overcome these difficulties has been observed in the literature and there is a consensus about the central role of regular faculty development programmes[4,16,21,22].

## Conclusion

The social representations theory was instrumental to show the experience of students and tutors rather than the satisfaction and "happiness factor" often reported in other literature. Faculty training on PBL tutorials and the evaluation of new programmes is absolutely necessary and it is envisaged that planners can make better-informed decisions about curricular implementation, review and reform when information of this nature is made available to them.

## Competing interests

The authors declare that they have no competing interests.

## Authors' contributions

VRB conceived the study, participated in its design and coordination, contributed to the analyses and drafted the manuscript. MHCG and CC participated in the study design, interviews and coordination. RCPL conceived the study design and was responsible for the discourse analyses (results) and contributed to draft the manuscript. All authors read and approved the final manuscript.

## Pre-publication history

The pre-publication history for this paper can be accessed here:


